# *APOE* genotype dictates lipidomic signatures in primary human hepatocytes

**DOI:** 10.1016/j.jlr.2024.100498

**Published:** 2024-01-11

**Authors:** Francisco C. Almeida, Kalicharan Patra, Andreas Giannisis, Anezka Niesnerova, Renu Nandakumar, Ewa Ellis, Tiago Gil Oliveira, Henrietta M. Nielsen

**Affiliations:** 1 Life and Health Sciences Research Institute (ICVS), School of Medicine, University of Minho, Braga, Portugal; 2 ICVS/3B’s—PT Government Associate Laboratory, Braga/Guimarães, Portugal; 3 Department of Neuroradiology, Centro Hospitalar Universitário do Porto, Porto, Portugal; 4 Department of Biochemistry and Biophysics, Stockholm University, Stockholm, Sweden; 5 Irving Institute for Clinical and Translational Research, Columbia University Irving Medical Center, New York, USA; 6 Department of Clinical Science, Intervention and Technology, (CLINTEC), Division of Transplantation surgery, Karolinska Institutet and ME Transplantation, Karolinska University Hospital, Huddinge, Sweden; 7 Department of Neuroradiology, Hospital de Braga, Braga, Portugal

**Keywords:** Apolipoprotein E, acylcarnitines, Alzheimer’s disease, genetic risk factor, hepatic lipids

## Abstract

Apolipoprotein E (*APOE*) genetic variants are most notably known for their divergent impact on the risk of developing Alzheimer’s disease. While *APOE* genotype has been consistently shown to modulate lipid metabolism in a variety of cellular contexts, the effect of *APOE* alleles on the lipidome in hepatocytes is unknown. In this study, we investigated the contribution of *APOE* alleles to lipidomic profiles of donor-derived primary human hepatocytes from 77 subjects. Lipidomic data obtained by liquid chromatography-mass spectrometry were analyzed across ε2/ε3, ε3/ε3, and ε3/ε4 genotypes to reveal how *APOE* modulates lipid relative levels over age and between groups. Hepatic APOE concentration, measured by ELISA, was assessed for correlation with lipid abundance in subjects grouped as per *APOE* genotype and sex. *APOE* genotype-specific differential lipidomic signatures associated with age for multiple lipid classes but did not differ between sexes. Compared to ε2/ε3, ε3/ε4 hepatocytes had higher abundance of acylcarnitines (AC) and acylphosphatidylglycerol (AcylPG) as a class, as well as higher medium and long-chain ACs, AcylPG, phosphatidylglycerol (PG), bis(monoacylglycerol)phosphate (BMP), monoacylglycerol (MG) and diacylglycerol (DG) species. The *ε*3/*ε*4 hepatocytes also exhibited a higher abundance of medium and long-chain ACs compared to the *ε*3/*ε*3 hepatocytes. Only in the *ε*3/*ε*4 hepatocytes, APOE concentration was lower and showed a negative correlation with BMP levels, specifically in females. *APOE* genotype dictates a differential lipidome in primary human hepatocytes. The lipids involved suggest mitochondrial dysfunction with accompanying alterations in neutral lipid storage, reflective of a general disturbance of free fatty acid metabolism in human hepatocytes with the *ε*4 allele.

Apolipoprotein E (APOE) in humans is produced predominantly by the liver and to a lesser degree by cells in the brain (1). Pertaining to its role in lipid metabolism and cholesterol homeostasis (2), *APOE* is the strongest genetic risk factor for Alzheimer’s disease (AD) (3) and has been implicated in several metabolic (4, 5, 6), cardiovascular (7) and other neurodegenerative diseases (8, 9, 10, 11). The three common *APOE* alleles, ε2, ε3, and ε4, only found in humans, give rise to three isoforms (APOE2, E3, and E4) that arise from single amino acid substitutions at positions 112 and 158 in the receptor binding domain of the mature protein, and result in defective receptor binding ability of E2, and a cross-domain interaction that affects lipoprotein preference by E4 (12). Owing to their structural variations, ε2 homozygosity contributes to familial dysbetalipoproteinemia (13), the presence of the ε4 allele increases the risk of atherosclerosis and late-onset AD (14), whereas the ε3 allele is considered as AD-risk neutral.

As APOE does not cross the blood-brain barrier (1), the trend in the scientific community has been to focus investigations on the role of APOE protein levels and gene polymorphisms separately in the peripheral (2, 7) and central nervous system compartments (15, 16, 17). However, recent findings have contributed to a paradigm shift to also consider plasma APOE levels, and specifically hepatic APOE in the context of neurodegenerative disorders (18, 19, 20). Peripheral APOE levels and isoform composition have been linked to brain metabolic processes and AD-related brain pathology using clinical cohorts (21, 22, 23, 24) and humanized liver mice (18).

Hepatocytes produce APOE (1) as part of various lipoprotein particles to participate in exogenous as well as endogenous lipoprotein pathways (25, 26). Apolipoprotein E is a key player in maintaining the balance between the in-flux and eflux of lipids in the liver, and a dysregulation may cause fatty acids, primarily integrating glycerolipids, such as triglycerides (TGs), to accumulate in hepatocytes resulting in steatohepatitis, cirrhosis and hepatic carcinoma (27). This dependency of hepatocytes on APOE raises an important question of exploring *APOE* genotype-specific hepatic lipid signatures.

Age-related changes in cholesterol metabolism appear to occur in the liver and the brain simultaneously (28). Nonalcoholic fatty-liver disease (NAFLD), a disorder of hepatic cholesterol metabolism (29), is considered a risk factor for AD (30). Apolipoprotein E deficiency is known to promote NAFLD in mice lacking *Apoe* (31) and ε4 was found to be overrepresented in NAFLD patients in a case-controlled study (32). In humans, represented by a case study of the complete absence of APOE, very high levels of plasma cholesterol and TG were observed and mostly distributed in large lipoparticles such as very low-density lipoprotein (VLDL) and intermediate-density lipoprotein IDL (33). The APOE-deficient patient, aged 40 years, had severe xanthomas in various organs but normal neurocognitive and cardiac functions, which may suggest that the presence of APOE4 rather than the absence of APOE, may promote cognitive dysfunction. These findings build a strong rationale for focusing on hepatic lipidomics in the context of liver disease, dementia, and aging. Lipidomic studies have already been very informative to fatty-liver diseases (34, 35, 36, 37, 38) and neurodegenerative diseases (39, 40, 41, 42). However, certain discrepancies in the results of lipidomic analyses in human and mouse models could be attributed to inter-species profile differences in lipoprotein particles. Such potential differences in lipoprotein composition between humans and animal models tend to complicate the translation of results from one to another. In the current study, we have assessed lipidomic profiles of primary human hepatocytes in order to identify *APOE* genotype-specific intracellular lipid signatures.

## Materials and Methods

### Primary human hepatocytes

Primary human hepatocytes were isolated using a previously established protocol that yields up to 97% purity (43), from a total of 77 donor-derived liver tissues acquired through liver transplantation or resection in the Liver Cell Laboratory at the Karolinska Institute, Stockholm, Sweden between the years 2016–2020. Briefly, liver tissue was first flushed with cold sterile Hank’s balanced salt solution (HBSS) supplemented with 10 mM 4-(2-hydroxyethyl)-1-piperazine ethane sulfonic acid (HEPES) and then consecutively perfused with three different solutions at 37°C; 1) 500 ml HBSS without Ca^2+^ and Mg^2+^ supplemented with 10 mM HEPES and 5 mM EGTA for 10 min, 2) 500 ml HBSS with Ca^2+^ and Mg^2+^ supplemented with 10 mM HEPES for 10 min, and 3) 1000 ml Eagle’s minimum essential medium (EMEM) supplemented with 250 mg collagenase XI and 50 mg DNase for approximately 24–27 min until the tissue disintegrated. Cold PlasmaLyte was added to the resulting cell suspension and the cell suspension was filtered through three layers of sterile gauze. The filtered suspension was cleaned up by low-speed centrifugation at 79 *g*, at 4°C for 5 min, and resuspended in cold PlasmaLyte. Following another round of centrifugation, the cells were re-suspended in Williams E medium. The cell suspension was finally centrifuged again, the supernatant discarded and the remaining cell pellet snap-frozen in liquid nitrogen and stored at −80°C until use. The collection of liver tissues and isolation of primary human hepatocytes from de-identified subjects was approved by the Regional Ethics Committee in Stockholm (2010/678-31/3 and 2017/269-31). The study was conducted in compliance with the Helsinki Declaration.

### *APOE* genotyping

Determination of *APOE* genotype was carried out essentially as previously described (24). In brief, DNA, extracted using the DNeasy® Blood & Tissue kit (QIAGEN), was used for *APOE* genotyping by TaqMan® SNP genotyping assays targeting the rs429358 and rs7412 variants of the *APOE* gene (ThermoFisher Scientific). Genotype was determined by a combination of results from both assays (rs429358 and rs7412).

### Quantification of hepatic APOE

Hepatic APOE content was measured by a previously described enzyme-linked immunosorbent assay (ELISA) (23). Briefly, primary human hepatocyte lysates were prepared in standard radioimmunoprecipitation assay (RIPA) buffer containing protease and phosphatase inhibitors (ThermoFisher Scientific), and the total protein content was determined by the Pierce bicinchoninic acid (BCA) assay (ThermoFisher Scientific). Apolipoprotein E was quantified in lysates by a sandwich ELISA using a mouse monoclonal anti-APOE (Novus Biologicals) antibody as the capture antibody and a biotinylated goat polyclonal anti-APOE (Meridian) antibody as the detection antibody. Levels of APOE were calculated by interpolation from a standard curve based on the optical density measured at 450 nm in the HiPo-96 microplate photometer (BIOSAN, Riga, Latvia), and normalized to the total protein content (ng APOE/mg total protein).

### Lipidomic analysis

Lipidomic profiling was performed using Ultra Performance Liquid Chromatography-Tandem Mass Spectrometry (UPLC-MSMS) at the Biomarkers Core Laboratory (Irving Institute for Clinical and Translational Research, Columbia University Medical Center). The applied assay detects 593 lipid species belonging to a total of 34 lipid classes (44). Lipid extracts were prepared from hepatocyte lysates spiked with appropriate internal standards using a modified Bligh and Dyer method (45), and analyzed on a platform comprising Agilent 1260 Infinity HPLC integrated to Agilent 6490A QQQ mass spectrometer controlled by Masshunter v 7.0 (Agilent Technologies). Glycerophospholipids and sphingolipids were separated with normal-phase HPLC as described before (46), with a few modifications. An Agilent Zorbax Rx-Sil column (2.1 × 100 mm, 1.8 μm) maintained at 25°C was used under the following conditions: mobile phase A (chloroform: methanol: ammonium hydroxide, 89.9:10:0.1, v/v) and mobile phase B (chloroform: methanol: water: ammonium hydroxide, 55:39:5.9:0.1, v/v); 95% A for 2 min, decreased linearly to 30% A over 18 min and further decreased to 25% A over 3 min, before returning to 95% over 2 min and held for 6 min. Separation of sterols and glycerolipids was carried out on a reverse phase Agilent Zorbax Eclipse XDB-C18 column (4.6 × 100 mm, 3.5um) using an isocratic mobile phase, chloroform, methanol, 0.1 M ammonium acetate (25:25:1) at a flow rate of 300 μl/min. Quantification of lipid species was accomplished using multiple reaction monitoring (MRM) transitions (46, 47, 48) under both positive and negative ionization modes in conjunction with referencing of appropriate internal standards: phosphatidic acid (PA) 14:0/14:0, phosphatidylcholine (PC) 14:0/14:0, phosphatidylethanolamine (PE) 14:0/14:0, phosphatidylglycerol (PG) 15:0/15:0, phosphatidylinositol (PI) 17:0/20:4, phosphatidylserine (PS) 14:0/14:0, bis-monoacylglycerol phosphate (BMP) 14:0/14:0, acyl phosphatidylglycerol (AcylPG) 14:0/14:0, LPC 17:0, lysophosphatidylethanolamine (LPE) 14:0, lysophosphatidylinositol (LPI) 13:0, ceramide (Cer) d18:1/17:0, sphingomyelin (SM) d18:1/12:0, dihydrosphingomyelin (dhSM) d18:0/12:0, galactosylceramide (GalCer) d18:1/12:0, glucosylceramide (GluCer) d18:1/12:0, lactosylceramide (LacCer) d18:1/12:0, D7-cholesterol, cholesterol ester (CE) 17:0, monoacylglycerol (MG) 17:0, 4 ME 16:0 diether diacylglycerol (DG), D5-TG 16:0/18:0/16:0 (Avanti Polar Lipids, Alabaster, AL). Lipid levels for each sample were calculated by summing up the total number of moles of all lipid species measured by all three LC-MS methodologies and then normalizing that total to mol %. Lipid classes and lipid species that did not reliably reach the detection limit due to low abundance, such as N-acylphosphatidylethanolamine (NAPE) and N-acylphosphatidylserine (NAPS), were excluded from further analyses. Out of a total of 593 analyzed lipid species, a total of 46 were excluded from the analysis due to low/undetectable content. The final data are presented as mean mol % with error bars showing mean ± S.D.

### Statistics

Lipid data (mol %) was assessed for normal distribution by the Shapiro-Wilk test for goodness of fit. A total of 23 out of 32 classes of lipids were found to have a skewed distribution. Therefore, the data was log2-transformed, z-scored, and correlated with age using the Pearson’s coefficient test. For *APOE* group comparisons, lipid abundance was corrected for age and sex, and compared using one-way ANOVA with Tukey HSD correction. Fold change was calculated from the means of the residuals for each group comparison and the logarithm of 2 was applied for volcano plot construction. Statistical significance was considered for *P*-values ≤ 0.05. Using R function *prcomp*, principal component analysis was conducted on lipid class and species abundance residuals after age and sex correction scaled to unit variance and centered. R package *factoextra* was used for data visualization. All statistical analyses and figures were created using RStudio 2021.09.1+372. Using the JMP Pro 16 statistical software, hepatic APOE concentrations were first compared between *APOE* genotype groups and sexes by use of the Wilcoxon test and then correlated with age and individual lipid classes using the Spearman’s rho test. The predictive value of APOE concentrations for levels of any lipid class was assessed by a linear regression model comparing log-transformed values for APOE and lipids.

## Results

### Study cohort demographics and clinical characteristics

Our cohort included 77 subjects: 45 *APOE* ε3/ε3, 12 *APOE* ε2/ε3, 17 *APOE* ε3/ε4, 1 *APOE* ε2/ε2, 1 *APOE* ε2/ε4 and 1 *APOE* ε4/ε4. Hence, out of the consecutively collected donor-derived tissues, a total of 25% of donors carried the ε4 allele. Given the low sample number of *APOE* ε2/ε2, ε2/ε4, and ε4/ε4 (n=1, each), we excluded samples from these donors from the statistical analyses (data is instead reported as observations). Data analysis was therefore performed on three groups in order to test for the effects of having either one protective allele, ε2, or one AD risk allele, ε4, compared to the neutral AD-risk condition of having two ε3 alleles.

In the 74 included subjects, there were no statistically significant differences regarding demographic characteristics between the *APOE* genotype groups (Table 1). Demographic data for excluded subjects are presented in supplemental Table S1. The analysis was conducted on 547 lipid species belonging to 32 classes. We found Free cholesterol (FC), TG, and PE to be the most abundant lipids, whereas AcylPG, ether lysophosphatidylcholine (LPCe), and N-acyl serine (NSer) to be the least abundant in the hepatocytes (supplemental Table S2). The clinical indication for liver resection or transplantation for the included subjects is presented in supplemental Table S3.

**Table 1:** Study participant summary demographics

Cohort (n = 74)	ε3/ε3 (n = 45)	ε2/ε3 (n = 12)	ε3/ε4 (n = 17)	*P*-value
Male (%)	24 (53%)	5 (42%)	10 (59%)	*X*(2) = 0.849; *P* = 0.654
Mean Age (±std)	54 (±24)	58 (±23)	65 (±18)	*F*(2) = 1.380; *P* = 0.258

Summary demographics for study participants according to *APOE* genotype. Std, standard deviation.

### Hepatic lipid abundance is modulated by age and *APOE* genotype

**Fig. 1:**
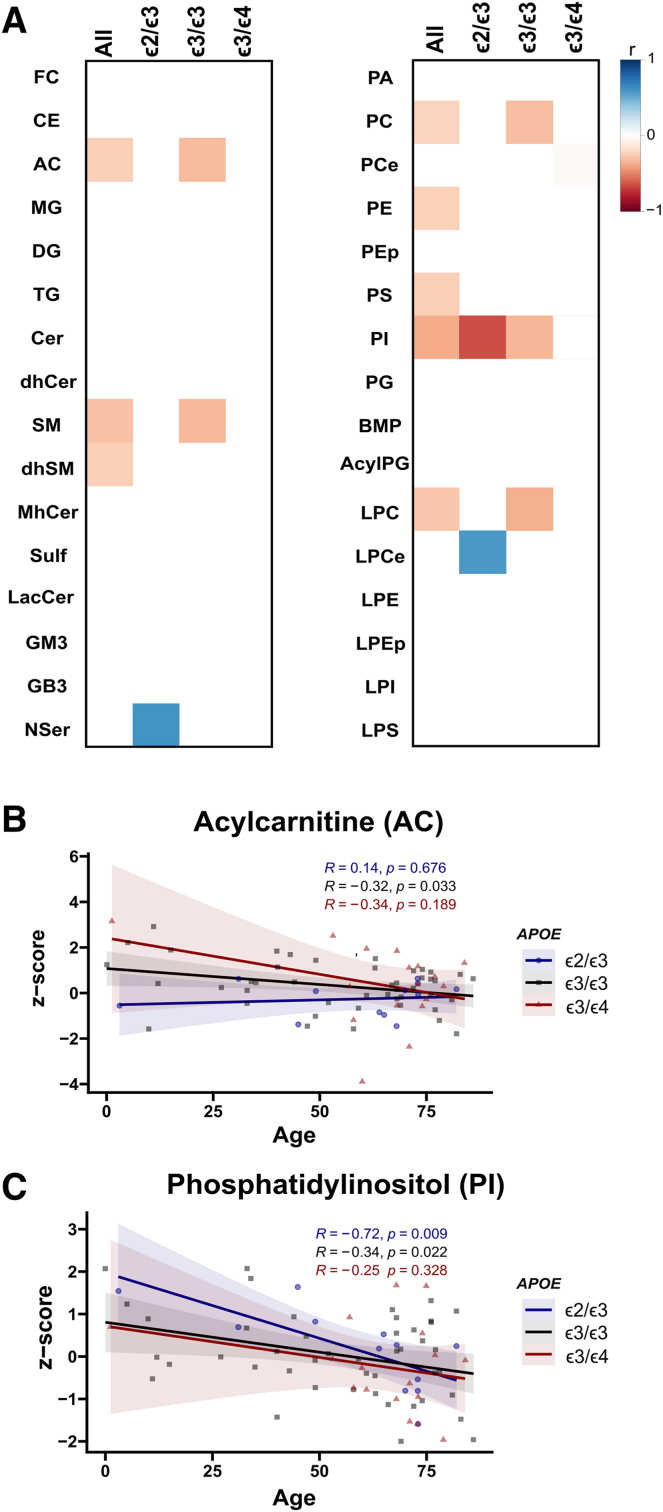
Lipid class abundance changes over age. Lipid class z-score Pearson’s correlation with age across the whole sample and within each group. Colored boxes in the heatmap represent Pearson’s r coefficient for statistically significant correlations determined as *P*-value ≤ 0.05 (A); Correlation plot for AC across *APOE* genotypes (B); Correlation plot for PI across *APOE* genotypes (C). AcylPG, Acylphosphatidylglycerol; AC, Acyl carnitine; BMP, Bis(monoacylglycero)phosphate; Cer, Ceramide; CE, Cholesterol ester; dhCer, Dihydroceramide; dhSM, Dihydrosphingomyelin; DG, Diacylglycerol; FC, Free cholesterol; GB3, Globotriaosylceramide; GM3, Monosialodihexosylganglioside; MG, Monoacylglycerol; LPEp, Plasmogen Lysophosphatidylethanolamine; LacCer, Lactosylceramide; LPI, Lysophosphatidylinositol; LPS, Lysophosphatidylserine; LPCe, Ether lysophosphatidylcholine; LPC, Lysophosphatidylcholine; LPE, Lysophosphatidylethanolamine; MHCer, Monohexosylceramide; NSer, N-Acyl Serine; PA, Phosphatidic acid; PC, Phosphatylcholine; PCe, Ether phosphatidylcholine; PE, Phosphatidylethanolamine; Pep, Plasmalogen phosphatidylethanolamine; PI, Phosphatidylinositol; PG, Phosphatidylglycerol; PS, Phosphatidylserine; SM, Sphingomyelin; Sulf, Sulfatide; TG, Triacylglycerol.

**Fig. 2:**
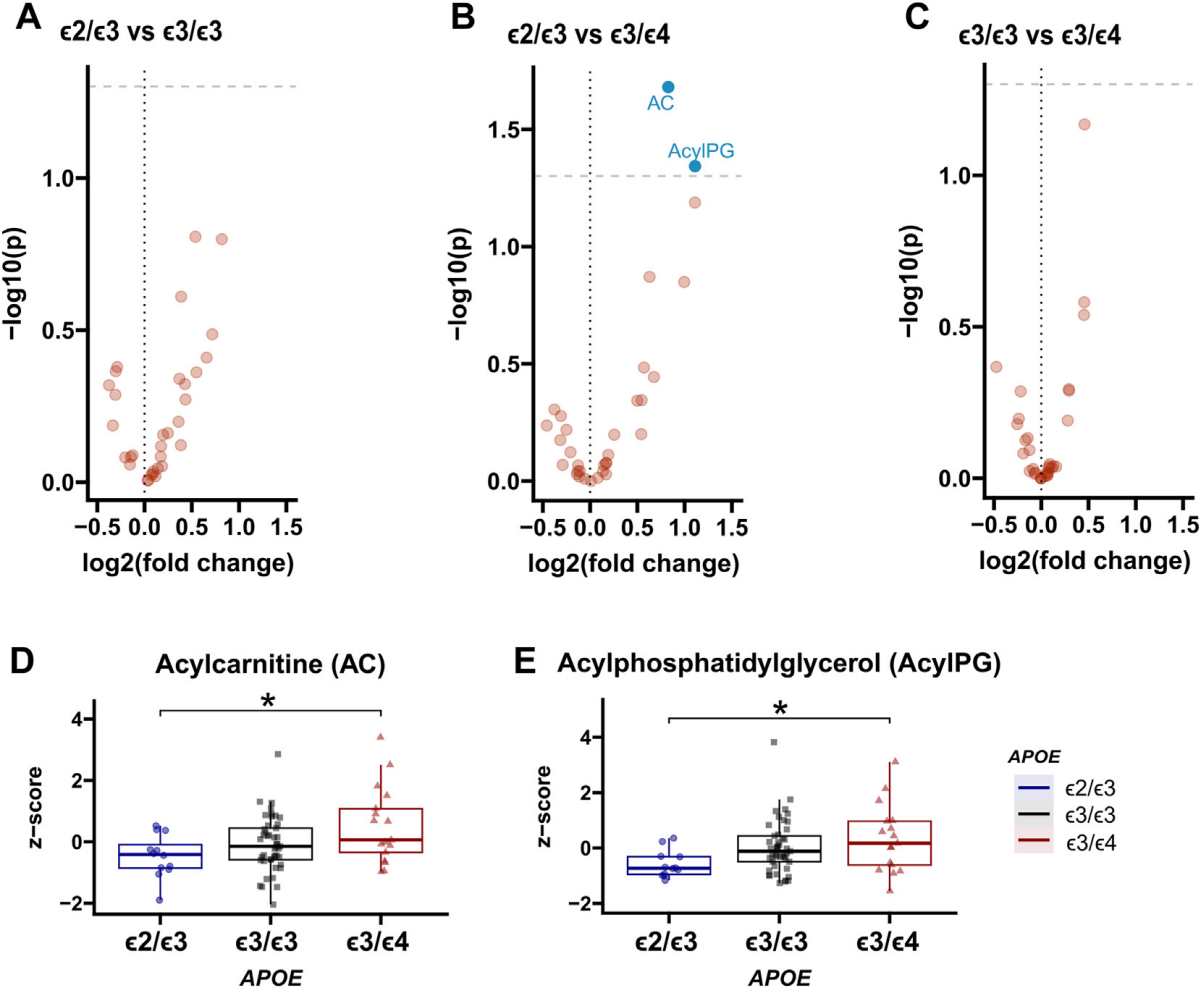
Lipid class abundance depends on *APOE* genotype. Volcano plots for lipid class relative abundance comparisons across *APOE* genotypes. *APOE* ε2/ε3 versus *APOE* ε3/ε3 (A); *APOE* ε2/ε3 versus *APOE* ε3/ε4 (B); *APOE* ε3/ε3 versus ε3/ε4 (C); Boxplot for AC across *APOE* genotypes (D); Boxplot for AcylPG across *APOE* genotypes (E); blue dots represent statistically significant comparisons after one-way ANOVA with TukeyHSD for multiple comparisons. ∗ - *P*-value ≤ 0.05.

**Fig. 3:**
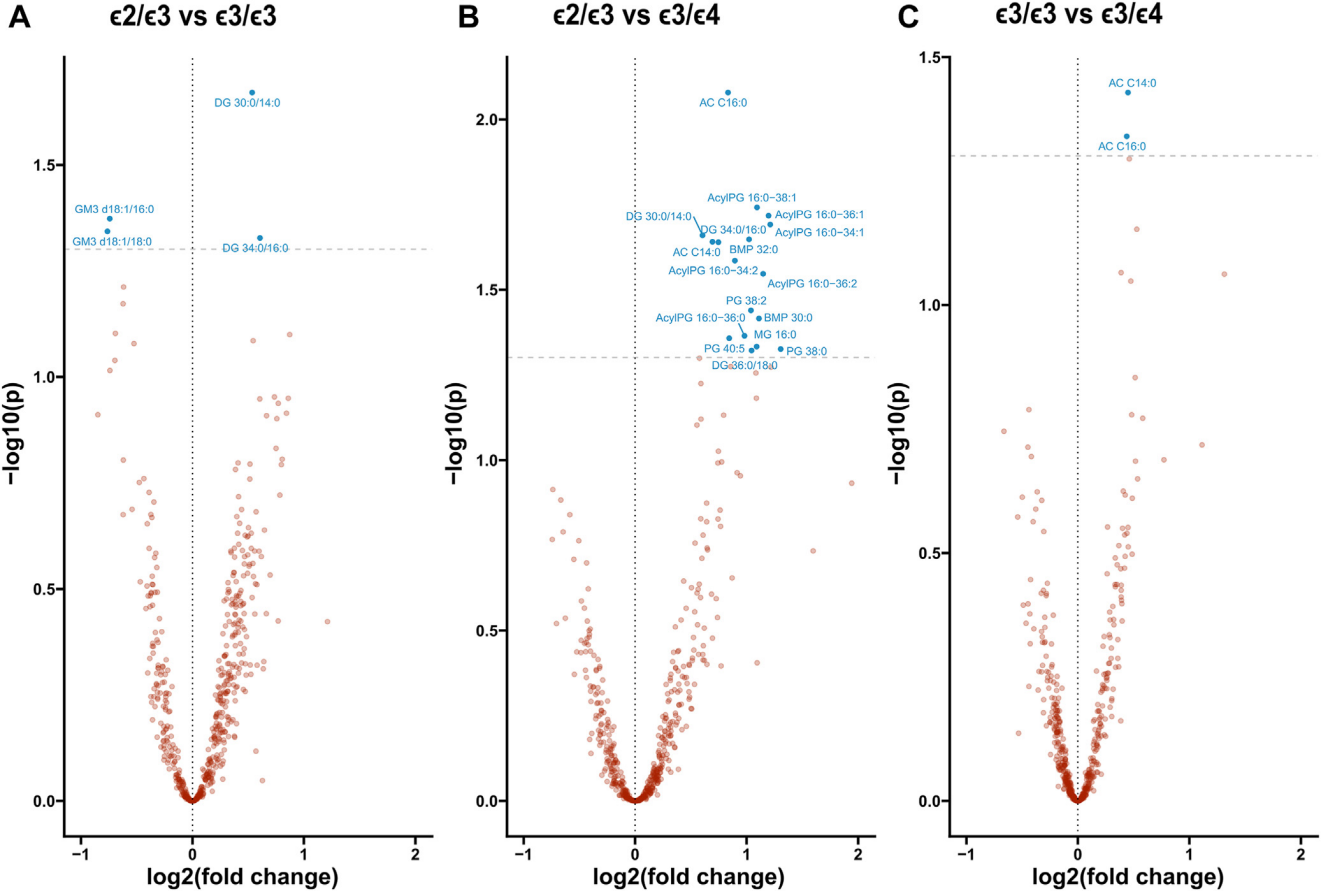
Lipid species abundance depends on *APOE* genotype. Volcano plots for lipid species comparisons across *APOE* genotypes. *APOE* ε2/ε3 versus *APOE* ε3/ε3 (A); *APOE* ε2/ε3 versus *APOE* ε3/ε4 (B); *APOE* ε3/ε3 versus ε3/ε4 (C); blue dots represent statistically significant comparisons after one-way ANOVA with Tukey HSD for multiple comparisons. AcylPG, Acylphosphatidylglycerol; AC, Acyl carnitine; BMP, Bis(monoacylglycero)phosphate; DG, Diacylglycerol; GM3, Monosialodihexosylganglioside; MG, Monoacylglycerol; PG, Phosphatidylglycerol.

We next analyzed how lipid classes may be modulated by age and sex. There were bidirectional associations with age across lipid classes and *APOE* groups (Fig. 1). Comparison of lipid abundance levels between sexes, or pairwise comparison across *APOE* genotypes and sex combined, did not reveal statistically significant differences for any lipid class (supplemental Tables S4 and S5). Assessing the abundance of lipid classes between *APOE* genotypes, we found higher relative levels of acyl carnitine (AC) and AcylPG in the *ε*3/*ε*4 group versus the *ε*2/*ε*3 (*P*-value ≤ 0.05; Fig. 2). When comparing the abundance of lipid species between *APOE* genotype groups (Fig. 3), we further found that the *ε*2/*ε*3 group displayed lower relative levels of monosialodihexosylganglioside (GM3) d18:1/16:0 and GM3 d18:1/18:0, and higher relative abundance of DG 30:0/14:0 and DG 30:0/16:0 versus the *ε*3/*ε*3 group (*P*-value ≤ 0.05). Higher relative abundance of AC C14:0, AC C16:0, AcylPG 16:0−34:1, AcylPG 16:0−34:2, AcylPG 16:0−36:0, AcylPG 16:0−36:1, AcylPG 16:0−36:2, AcylPG 16:0−38:1, PG 38:0, PG 38:2, PG 40:5, MG 16:0, DG 30:0/14:0, DG 34:0/16:0, DG 34:0/18:0, BMP 30:0 and BMP 32:0 were in turn found in the ε3/ε4 group compared to the ε2/ε3 group (*P*-value ≤ 0.05). Finally, higher relative levels of AC C14:0 and AC C16:0 were found in the *ε*3/*ε*4 hepatocytes compared to *ε*3/*ε*3 hepatocytes (*P*-value ≤ 0.05) (Fig. 3).

Next, we used principal component analysis (PCA) to assess the variation of lipid classes and species due to *APOE* genotype. Using residuals after age and sex correction, among lipid classes, the first and second principal components explained 57% of the variance. Using lipid species abundance after age and sex correction, the first and second principal components explained 43% of the variance. In both situations, the PCA did not clearly separate *APOE* groups (supplementary Fig. S2).

### Hepatic APOE content is influenced by *APOE* genotype and negatively correlates with age and specific lipid classes

**Fig. 4:**
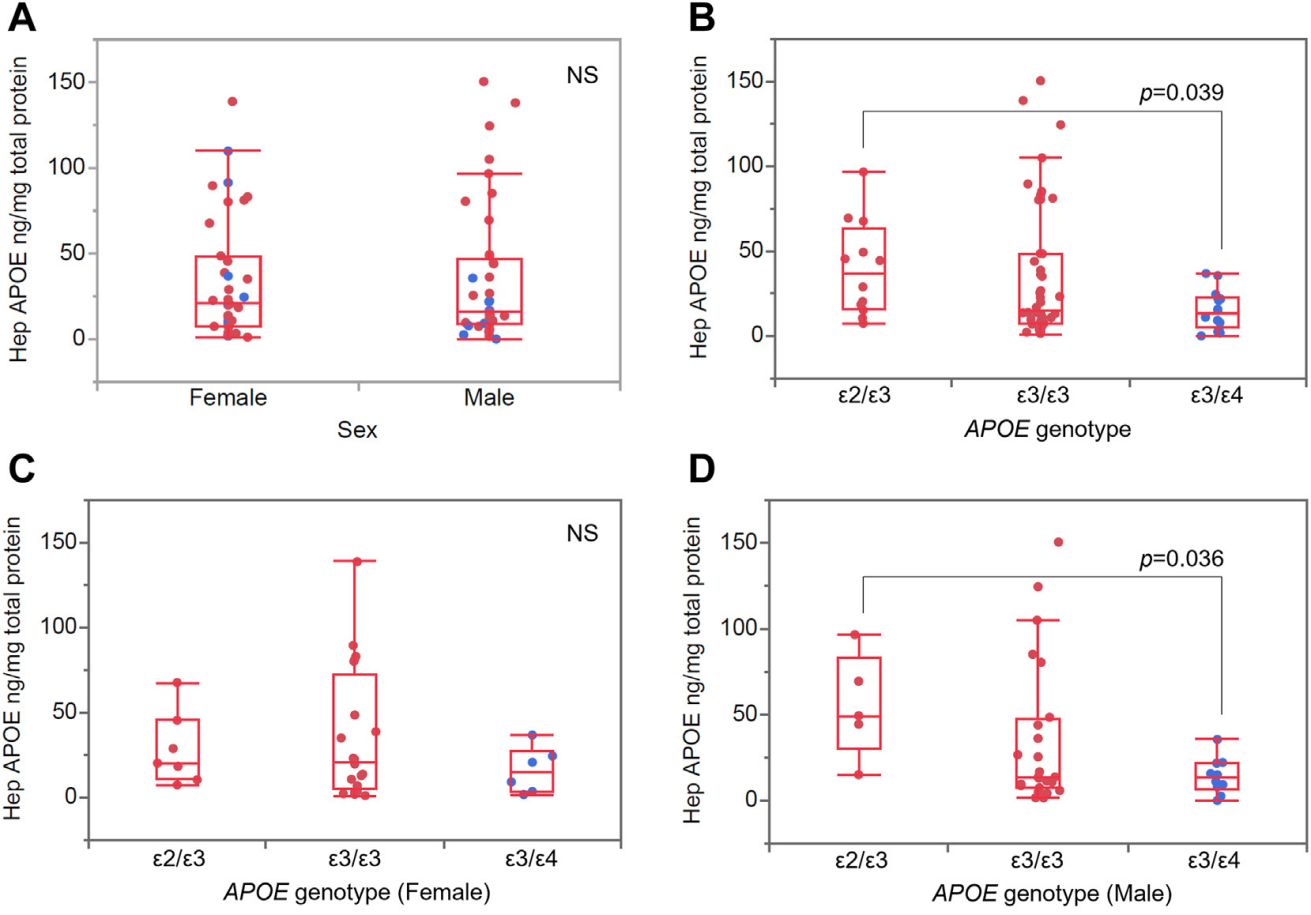
Hepatic APOE content according to sex and *APOE* genotype. Hepatic APOE content compared between sexes (A) and between *APOE* genotypes (B), and between females (C) and males (D) grouped as per *APOE* genotype. NS: no significant difference. blue dot: ε4 carrier; red dot: ε4 non-carrier.

**Fig. 5:**
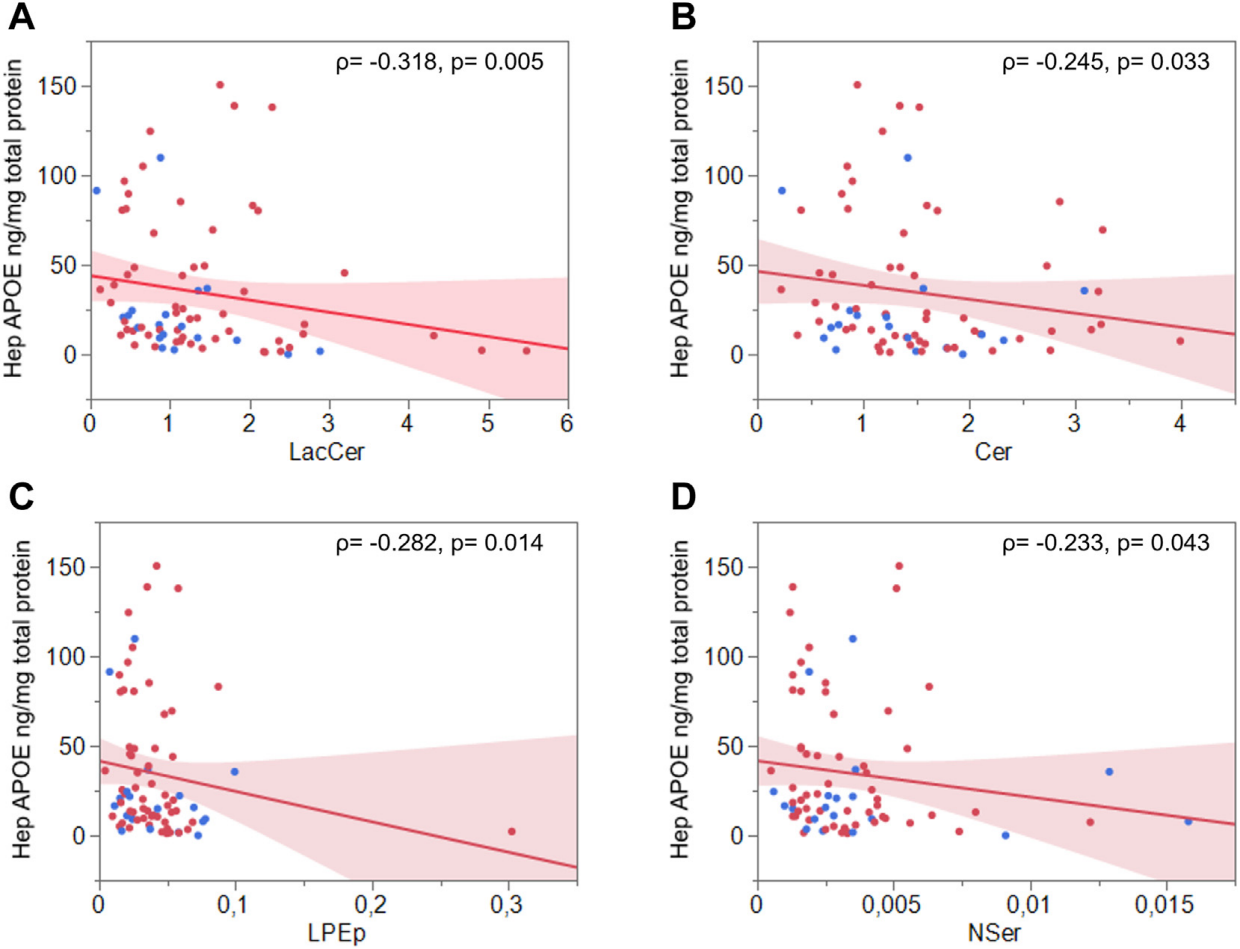
Association between hepatic APOE content and relative lipid levels. Significant associations between hepatic APOE content (ng/mg total protein) and lipid class relative abundance- LacCer (A), Cer (B), LPEp (C) and NSer (D). ρ: Spearman’s correlation coefficient, p: prob [ρ]. *APOE ε*4 carriers are marked as blue dots, and non-carriers as red dots.

We further analyzed hepatic APOE content (ng APOE per mg total protein) by stratifying the groups based on sex and ε4 status. The APOE concentrations ranged between 0.1 ng/mg to 150.5 ng/mg (median 19.1 ng/mg; 75% quartile 47.75 ng/mg, 25% quartile 8.825 ng/mg). We did not find any significant difference in hepatic APOE content between the sexes (Fig. 4A), however comparing APOE content between the three *APOE* genotype groups (*ε*2/*ε*3, *ε*3/*ε*3, and *ε*3/*ε*4), hepatic APOE concentrations were 2.8-fold higher in hepatocytes derived from *ε*2/*ε*3 (n = 12) compared to *ε*3/*ε*4 (n = 16, one outlier was removed) donors (*P* = 0.039, after Bonferroni correction) (Fig. 4B). Interestingly, male *ε*2/*ε*3 hepatocytes (n = 5) exhibited more than two-fold higher APOE content, although statistically not significant due to large variation, compared to female *ε*2/*ε*3 hepatocytes (n = 7). Hence, the statistically significant difference in hepatic APOE content between *ε*2/*ε*3 and *ε*3/*ε*4 hepatocytes was not evident in hepatocytes from female *ε*3/*ε*4 donors (Fig. 4C, D). We further observed a negative correlation between hepatic APOE levels and the age of the donor (ρ = −0.29, *P* = 0.01), which was lost when subjects were grouped as per sex or *APOE* genotype. We also assessed potential correlations between hepatic APOE content and individual lipid classes (mol%) first in the whole cohort, and then in three *APOE* genotype groups. In the whole cohort, we observed negative correlations between hepatic APOE content and the abundance of Cer, LacCer, LPEp, and NSer (Fig. 5). However, when subjects were grouped according to *APOE* genotype, the correlation only remained in the 3/*ε*3 group (supplemental Table S6). Furthermore, only the 3/*ε*4 genotype group (*APOE*ε4-carriers) exhibited a significant negative correlation between hepatic APOE and BMP (ρ = −0.64, *P* = 0.01).

### Hepatic APOE has predictive value for lipid classes

To assess whether APOE levels have any predictive value for the abundance of any of the measured lipids, linear regression was performed on log-transformed values for APOE and lipids, first in the whole cohort and then after grouping per genotype and sex. In the whole cohort, APOE showed a negative association with Cer (r = −0.24), LacCer (r = −0.41), BMP (r = −0.24), LPEp (r = −0.34), and NSer (r = −0.25) (*P*-value ≤ 0.05), which was lost when the cohort was divided into genotype groups; except for LacCer, which was driven by ε3/ε3 (r = −0.40, *P* = 0.01) and ε3/ε4 (r = −0.57, *P* = 0.02) subjects, and for LPEp, which was only driven by ε3/ε3 (r = −0.33, *P* = 0.02). Interestingly, for BMP, only female ε3/ε4 subjects appeared to drive the association seen at the whole cohort level (r = −0.80, *P* = 0.05). However, in the case of Cer and NSer, the association was sustained only in females but not in any *APOE* genotype groups (Cer: r = −0.37, *P* = 0.03; NSer: r = −0.39, *P*= 0.02. Additionally, APOE correlated with dhCer only in female subjects (r = −0.33, *P* = 0.05).

## Discussion

We herein describe differential effects of *APOE* genotype on the lipidome and APOE content of primary human hepatocytes, consecutively collected during the years 2016–2020 at the Karolinska Institute in Stockholm, Sweden. The cells were snap-frozen upon isolation and never cultured, hence the hepatocytes did not undergo any potential cellular alterations, including de-differentiation (49) as a consequence of in vitro culture conditions. Therefore, the cellular lipid and APOE content is representative of the content in vivo. A total of 25% of the hepatocyte donors were ε4-carriers and only one out of 77 donors (less than 2%) was ε4 homozygous, suggesting that ε4 occurrence in the included donors is similar to that of the general population in Sweden (50).

We identified TG and glycerophospholipids to be among the most abundant lipids, comparable to results presented by a previous study on cultured primary human hepatocytes (51). Although the hepatic lipidome did not seem to differ between male and female donors, we found gradient effects of *APOE* genotype on lipid expression across age and at the lipid class level. *APOE*-genotype was associated with alterations in hepatic APOE content and in ε4-carriers, higher APOE levels were associated with lower levels of BMP.

*APOE* genotype was previously shown to affect lipid content in human plasma, cerebrospinal fluid, brain, and in stem-cell-derived astrocytes and cerebral organoids (40, 52, 53, 54, 55, 56, 57), as well as in the plasma and brain of genetically modified rodents (58, 59, 60, 61, 62, 63). Carrying the ε4 allele was in previous studies shown to modulate the effect of dietary fat intake on the risk of AD (64). Importantly, *APOE* has been shown to modulate the risk of AD in an age-dependent manner with the *APOE* ε4-induced higher risk of AD diminishing after 70 years of age (65). This *APOE* age-dependent modulation has also been shown for human plasma lipids, encompassing both PI and AC, among others (52), in agreement with our results on lipid abundance modulation by age. Specifically, AC species were differently modulated by age in the studied *APOE* groups, decreasing with age in ε3/ε3 (Fig. 1B), and found to be increased as a class in *APOE* ε3/ε4 versus ε2/ε3 (Fig. 2B, D). These differences were noted for medium to long-chain ACs (C14, C16) in ε3/ε4 versus ε2/ε3 and ε3/ε3. Acyl carnitines are intermediate metabolites involved in the transport of fatty acids across the mitochondrial membrane for β-oxidation. A range of enzymes are related to the processing of these metabolites according to their carbon chain, with different fatty acid oxidation disorders having a differential impact on the concentration of ACs of different lengths in tissue and plasma (66). Importantly, ε4 has been linked to higher amounts of plasma ACs in humans (67, 68) whereas lower plasma levels of short-chain ACs were associated with accelerated brain aging specifically in females (69). Furthermore, AC species were consistently found to be higher in AD and cognitively impaired patients (70, 71, 72, 73). In metabolic diseases with fatty acid oxidation defects (FAOD), it was proposed that mitochondrial dysfunction was caused by high tissue concentrations of fatty acids and AC, whereof long-chain species of the latter were described as toxic to the heart (74). These results further support the concept of altered AC levels and mitochondrial dysfunction related to the ε4 allele, and AD pathophysiology (75) with recent data also supporting the role for liver APOE in contributing to neurodegeneration (18, 19).

We in addition observed that human hepatocytes with an ε3/ε4 genotype had higher content of AcylPG as a class versus ε2/ε3 hepatocytes, as well as higher AcylPG, PG, BMP, MG, and DG species. While not much is known about the biological role of AcylPG, PG can be affected by AcylPG levels and is a proposed precursor for BMP (76). Interestingly, AcylPG was found to be the lipid class with the highest differential binding capacity to APOE2 when compared with APOE4 (77). Additionally, BMP is known to be present in late endosomes (78) and its accumulation has been linked with disturbances of the endolysosomal pathway in neurodegenerative disorders (79, 80). Since MG can be converted into DG, which can then further generate TG, an energy storage lipid and a main constituent of lipid droplets in cells, increased MG and DG levels suggest that lipid droplet formation is impaired in hepatocytes of ε4-carriers, alternatively that lipid droplet/mitochondria turnover is impaired due to lypophagy/mitophagy dysregulation in the context of endolysosomal malfunctioning. Interestingly, it was previously shown that DG levels were decreased in the entorhinal cortex of ε4 mice (58). While altered DG levels support the concept that the endolysosomal system in both the liver and brain cells is impaired, opposite observed effects could point to differential regulatory pathways. For instance, since ε4 has been shown to increase lipid droplet number and size in a variety of cells (56, 81, 82) and mitochondria-lipid droplet contacts are known to play roles in both lypolysis and lypogenesis (83, 84), higher MG and DG levels could be implicating APOE4 in processes related to fatty acid storage and utilization. We, therefore, speculate that more DG and MG *along with* AC might be the result of mitochondrial dysfunction with accompanying alterations in neutral lipid storage, reflective of a general disturbance of free fatty acid metabolism in human hepatocytes with an ε4 allele. On the other hand, hepatocytes with the ε2 allele in turn displayed higher abundance of two DG species in comparison to ε3 homozygous hepatocytes. More abundant DG could also suggest lower activity of DG lipases, alternatively enhanced DG formation by for example elevated activity of lipases (triglyceride) or phospholipases (phospholipids) (85). Previous studies have indeed shown altered DG lipase activity in AD (86), potentially related to the observed higher levels of DG in plasma from AD patients, indicative of membrane destabilization or breakdown (76). Importantly, whether the ε2 AD protective effect is mediated by opposing pathways driving the higher AD risk imposed by ε4, is not clear. In that respect, the DG 30:0/14:0 species was elevated in the comparison between both ε2/ε3 versus ε3/ε3 and ε2/ε3 versus ε3/ε4 hepatocytes. More studies are needed to elucidate whether alterations of specific DG species are indicative of dysfunction in specific organelle membranes and/or the outer cellular membrane, and whether alterations at the membrane level can be reflected in altered circulating DG levels. Moreover, hepatocytes with the ε2 allele displayed a relatively low abundance of two GM3 species in comparison to ε3 homozygous hepatocytes. The GM3 are membrane-bound glycosphingolipids, whose reduction was linked to improved insulin sensitivity (87, 88). We could not find any published data supporting the notion that specific GM3 species may be involved in the attenuation of insulin signaling, whereas there is ample literature suggesting insulin resistance, especially in ε4 carriers to be implicated in the development of AD (89). Therefore, we speculate that the lower levels of specific GM3 lipids in hepatocytes from ε2 carriers may exert a protective effect against AD via improved insulin signaling.

Apolipoprotein E in the periphery is mainly produced by the liver (1); however, it is also produced by other peripheral tissues such as monocytes, bone marrow, kidney, spleen, and skin (90, 91) which contributes to the plasma pool of APOE. Our data demonstrating nearly three-fold lower hepatic APOE content in hepatocytes from ε3/ε4 versus ε2/ε3-carriers is in line with previous reports of lower plasma APOE levels in ε4-carriers (23, 92, 93), although the difference in magnitude is larger in the here described intracellular hepatic APOE content compared to previously published differences in plasma APOE levels (92, 94, 95, 96). These results suggest that the plasma APOE content results from both intra- and extra-cellular metabolism of APOE. Also, we observed a slight decline in hepatic APOE levels with age, whereas plasma APOE was reported to increase with age (97). Intrahepatic APOE plays an important role in the synthesis and secretion of VLDL-TG lipoparticles without affecting the HDL pool (98), and as APOE4 could readily and preferably associate with VLDL because of its structural constraints (25), it could be assumed that hepatic APOE isoforms will have a differential effect on VLDL secretion from liver.

Interestingly, we noted that higher APOE levels were associated with lower levels of sphingolipids (Cer, LacCer, and dhSer), LPEp, NSer, and BMP. Sphingolipids have recently been implicated in cancer (99), metabolic disorders (100), as well as neurodegenerative diseases (101) with LacCer in particular related to inflammation in neurological disorders (102). Hence, that hepatic APOE levels may have predictive value for these sphingolipids is intriguing.

Our study has important limitations. We were not able to account for the effect of the different clinical diagnoses of the donors, some of which may be directly related to hepatic physiology although it is not clear how hepatocytes reflect on disease phenotype or stage when isolated. Moreover, *APOE* groups were not balanced in sample size with only one donor represented in the ε2/ε2, ε2/ε4, and ε4/ε4 groups, and our cohort included donors of a wide age range. Despite these shortcomings, this is to our knowledge the first study to address lipidomic signatures of *APOE* in primary human hepatocytes, opening new avenues for future studies.

Overall, our results support a role for *APOE* in modulating the hepatic lipidome and APOE content in an age-dependent manner. Altogether, these lipidomic signatures across *APOE* genotypes suggest a disturbance of mitochondrial metabolism and possibly fatty acid storage/utilization, as well as alterations of the endolysosomal system in the ε3/ε4 group (especially vs. ε2/ε3). Some of these effects might be age-dependent, concordant with the known risk effect of ε4 for AD (65). These results prompt further research into altered liver lipid metabolism in ε4-carriers and how it might impact brain metabolism and neurodegeneration.

## Data Availability

Data will be shared upon reasonable request to the corresponding author Henrietta M Nielsen.

## Supplemental data

This article contains supplemental data.

## Conflict of interest

The authors declare that they have no known competing financial interests or personal relationships that could have appeared to influence the work reported in this paper.
