# Supplementary material for: APOE genotype dictates lipidomic signatures in primary human hepatocytes
Source: J Lipid Res. 2024 Jan 11;65(2):100498. doi: 10.1016/j.jlr.2024.100498 (PMC10875595; doi:10.1016/j.jlr.2024.100498)

## ***APOE* genotype dictates lipidomic signatures in primary human hepatocytes**

Almeida F et.al.

**Supplementary Information**

**Supplementary Table 1 – Demographics of excluded *APOE* groups**

**Supplementary Table 2 – Lipid class relative abundance across *APOE* groups**

Relative abundance of lipid classes expressed as mol (%) for each *APOE* group (mean and standard deviation). * - Results for one-way ANOVA for the groups ε3/ε3, ε2/ε3 and ε3/ε4. FC - Free cholesterol; PCe - Ether phosphatidylcholine; CE - Cholesterol ester; PE - Phosphatidylethanolamine; AC - Acylcarnitine; Pep – Plasmalogen phosphatidylethanolamine; MG - Monoacylglycerol; PS - Phosphatidylserine; DG -Diacylglycerol; PI - Phosphatidylinositol; TG - Triacylglycerol; PG - Phosphatidylglycerol; dhCer - Dihydroceramide; BMP - Bis(monoacylglycero)phosphate; Cer - Ceramide; AcylPG - Acylphosphatidylglycerol; SM - Sphingomyelin; LPC - Lysophosphatidylcholine; dhSM - Dihydrosphingomyelin; LPCe - Ether lysophosphatidylcholine; Sulf - Sulfatide; LPE - Lysophosphatidylethanolamine; MHCer - Monohexosylceramide; LPEp - Plasmogen Lysophosphatidylethanolamine; LacCer - Lactosylceramide; LPI - Lysophosphatidylinositol; GM3 - Monosialodihexosylganglioside; LPS - Lysophosphatidylserine; GB3 - Globotriaosylceramide; PA - Phosphatidic acid; PC - Phosphatylcholine; NSer - N-Acyl Serine.

**Supplementary Table 3 - Number of donors per diagnosis**

Number of included donors per diagnosis. *Non-digestive system related* diagnosis pertains to patients who donated liver tissue after unrelated causes of death.

**Supplementary Table 4 – Lipid class comparisons between sexes across the whole sample**

P-values for sex group comparisons of lipid classes. There were no statistically significant findings. FC - Free cholesterol; PCe - Ether phosphatidylcholine; CE - Cholesterol ester; PE - Phosphatidylethanolamine; AC - Acylcarnitine; Pep – Plasmalogen phosphatidylethanolamine; MG - Monoacylglycerol; PS - Phosphatidylserine; DG -Diacylglycerol; PI - Phosphatidylinositol; TG - Triacylglycerol; PG - Phosphatidylglycerol; dhCer - Dihydroceramide; BMP - Bis(monoacylglycero)phosphate; Cer - Ceramide; AcylPG - Acylphosphatidylglycerol; SM - Sphingomyelin; LPC - Lysophosphatidylcholine; dhSM - Dihydrosphingomyelin; LPCe - Ether lysophosphatidylcholine; Sulf - Sulfatide; LPE - Lysophosphatidylethanolamine; MHCer - Monohexosylceramide; LPEp - Plasmogen Lysophosphatidylethanolamine; LacCer - Lactosylceramide; LPI - Lysophosphatidylinositol; GM3 - Monosialodihexosylganglioside; LPS - Lysophosphatidylserine; GB3 - Globotriaosylceramide; PA - Phosphatidic acid; PC - Phosphatylcholine; NSer - N-Acyl Serine.

**Supplementary Table 5 – Pairwise comparisons across *APOE* genotype and donor sex**

P-values for comparisons across *APOE* groups and sex after false-discovery rate correction for number of pairwise comparisons. M – male, F – female. FC - Free cholesterol; PCe - Ether phosphatidylcholine; CE - Cholesterol ester; PE - Phosphatidylethanolamine; AC - Acylcarnitine; Pep – Plasmalogen phosphatidylethanolamine; MG - Monoacylglycerol; PS - Phosphatidylserine; DG -Diacylglycerol; PI - Phosphatidylinositol; TG - Triacylglycerol; PG - Phosphatidylglycerol; dhCer - Dihydroceramide; BMP - Bis(monoacylglycero)phosphate; Cer - Ceramide; AcylPG - Acylphosphatidylglycerol; SM - Sphingomyelin; LPC - Lysophosphatidylcholine; dhSM - Dihydrosphingomyelin; LPCe - Ether lysophosphatidylcholine; Sulf - Sulfatide; LPE - Lysophosphatidylethanolamine; MHCer - Monohexosylceramide; LPEp - Plasmogen Lysophosphatidylethanolamine; LacCer - Lactosylceramide; LPI - Lysophosphatidylinositol; GM3 - Monosialodihexosylganglioside; LPS - Lysophosphatidylserine; GB3 - Globotriaosylceramide; PA - Phosphatidic acid; PC - Phosphatylcholine; NSer - N-Acyl Serine.

**Supplementary Table 6 – Correlation between hepatic APOE content and lipids**

Spearman’s rank correlation between hepatic APOE and relative abundance of all lipid classes in all subjects (N=74) excluding those of genotypes ε2/ε2 (1), ε2/ε4 (1), and ε4/ε4 (1). P-values for statistically significant correlations are marked in bold. FC - Free cholesterol; PCe - Ether phosphatidylcholine; CE - Cholesterol ester; PE - Phosphatidylethanolamine; AC - Acylcarnitine; Pep – Plasmalogen phosphatidylethanolamine; MG - Monoacylglycerol; PS - Phosphatidylserine; DG -Diacylglycerol; PI - Phosphatidylinositol; TG - Triacylglycerol; PG - Phosphatidylglycerol; dhCer - Dihydroceramide; BMP - Bis(monoacylglycero)phosphate; Cer - Ceramide; AcylPG - Acylphosphatidylglycerol; SM - Sphingomyelin; LPC - Lysophosphatidylcholine; dhSM - Dihydrosphingomyelin; LPCe - Ether lysophosphatidylcholine; Sulf - Sulfatide; LPE - Lysophosphatidylethanolamine; MHCer - Monohexosylceramide; LPEp - Plasmogen Lysophosphatidylethanolamine; LacCer - Lactosylceramide; LPI - Lysophosphatidylinositol; GM3 - Monosialodihexosylganglioside; LPS - Lysophosphatidylserine; GB3 - Globotriaosylceramide; PA - Phosphatidic acid; PC - Phosphatylcholine; NSer - N-Acyl Serine.

**Supplementary Figure 1 Lipid class abundance per *APOE* genotype and sex**

Boxplot for lipid class z-score of residuals derived from linear regression with age across *APOE* groups divided by sex. Wilcoxon tests were used with false discovery rate correction for pairwise multiple comparisons. F – female, M – male. FC - Free cholesterol; PCe - Ether phosphatidylcholine; CE - Cholesterol ester; PE - Phosphatidylethanolamine; AC - Acylcarnitine; Pep – Plasmalogen phosphatidylethanolamine; MG - Monoacylglycerol; PS - Phosphatidylserine; DG -Diacylglycerol; PI - Phosphatidylinositol; TG - Triacylglycerol; PG - Phosphatidylglycerol; dhCer - Dihydroceramide; BMP - Bis(monoacylglycero)phosphate; Cer - Ceramide; AcylPG - Acylphosphatidylglycerol; SM - Sphingomyelin; LPC - Lysophosphatidylcholine; dhSM - Dihydrosphingomyelin; LPCe - Ether lysophosphatidylcholine; Sulf - Sulfatide; LPE - Lysophosphatidylethanolamine; MHCer - Monohexosylceramide; LPEp - Plasmogen Lysophosphatidylethanolamine; LacCer - Lactosylceramide; LPI - Lysophosphatidylinositol; GM3 - Monosialodihexosylganglioside; LPS - Lysophosphatidylserine; GB3 - Globotriaosylceramide; PA - Phosphatidic acid; PC - Phosphatylcholine; NSer - N-Acyl Serine.

**Supplementary Figure 2 Principal component analysis of lipids per *APOE* genotype**

Scatter plot with the first and second principal components and scree plots for lipid classes (A-B) and species (C-D).

**Supplementary Table 1 – Demographics of excluded *APOE* groups**

|  | ε2/ε2 (n = 1) | ε2/ε4 (n = 1) | ε4/ε4 (n = 1) |
| --- | --- | --- | --- |
| Sex | Male | Female | Male |
| Age (years) | 71 | 60 | 74 |

**Supplementary Table 2 – Lipid class relative abundance across *APOE* groups**

| Lipid Class | ε3/ε3  (n = 45) | ε2/ε3  (n = 12) | ε3/ε4  (n = 17) | ε2/ε2  (n = 1) | ε2/ε4  (n=1) | ε4/ε4 (n=1) | *p-value** |
| --- | --- | --- | --- | --- | --- | --- | --- |
| FC | 21.07 (±14.46) | 27.57 (±21.54) | 22.80 (±16.44) | 17.16 | 13.06 | 28.26 | *F*(2,71) = 0.763; *p* = 0.470 |
| TG | 20.77 (±20.03) | 15.35 (±14.69) | 19.40 (±15.10) | 6.17 | 7.00 | 2.62 | *F*(2,71) = 0.419; *p* = 0.660 |
| PE | 15.82 (±7.32) | 18.79 (±9.47) | 15.31 (±7.87) | 27.7 | 29.15 | 26.79 | *F*(2,71) = 0.819; *p* = 0.445 |
| CE | 8.05 (±3.19) | 6.45 (±2.97) | 7.20 (±3.16) | 6.34 | 4.86 | 3.46 | *F*(2,71) = 1.400; *p* = 0.253 |
| PS | 4.39 (±1.79) | 4.24 (±1.02) | 3.82 (±1.71) | 6.45 | 7.10 | 3.87 | *F*(2,71) = 0.714; *p* = 0.493 |
| PC | 4.39 (±2.20) | 4.88 (±2.11) | 5.05 (±5.13) | 4.38 | 6.05 | 10.63 | *F*(2,71) = 0.334; *p* = 0.717 |
| PI | 3.84 (±1.76) | 4.08 (±1.74) | 3.37 (±1.88) | 4.03 | 5.10 | 6.80 | *F*(2,71) = 0.630; *p* = 0.536 |
| MG | 3.54 (±2.62) | 2.18 (±3.22) | 5.02 (±5.56) | 4.72 | 8.24 | 0.38 | *F*(2,71) = 2.280; *p* = 0.110 |
| SM | 2.53 (±1.02) | 2.71 (±0.69) | 2.38 (±1.21) | 2.57 | 3.34 | 4.76 | *F*(2,71) = 0.380; *p* = 0.685 |
| DG | 2.42 (±1.03) | 1.67 (±0.82) | 2.47 (±1.76) | 2.00 | 2.11 | 0.40 | *F*(2,71) = 2.016; *p* = 0.141 |
| BMP | 2.39 (±1.04) | 2.14 (±0.84) | 2.46 (±1.49) | 4.11 | 3.23 | 2.40 | *F*(2,71) = 0.287; *p* = 0.751 |
| PEp | 2.10 (±1.19) | 2.55 (±1.30) | 2.00 (±1.02) | 4.87 | 3.27 | 2.93 | *F*(2,71) = 0.891; *p* = 0.415 |
| AC | 1.61 (±1.33) | 0.93 (±0.43) | 2.19 (±2.28) | 1.19 | 0.69 | 1.10 | *F*(2,71) = 2.447; *p* = 0.094 |
| Cer | 1.58 (±0.83) | 1.36 (±0.88) | 1.38 (±0.71) | 1.53 | 1.41 | 0.77 | *F*(2,71) = 0.585; *p* = 0.560 |
| LacCer | 1.44 (±1.08) | 1.33 (±1.23) | 1.13 (±0.73) | 2.28 | 0.88 | 0.86 | *F*(2,71) = 0.542; *p* = 0.584 |
| dhSM | 1.30 (±0.51) | 1.15 (±0.61) | 1.30 (±0.58) | 1.54 | 0.95 | 0.87 | *F*(2,71) = 0.380; *p* = 0.685 |
| PCe | 0.78 (±0.35) | 0.76 (±0.26) | 0.80 (±0.49) | 0.77 | 1.28 | 1.35 | *F*(2,71) = 0.041; *p* = 0.960 |
| LPE | 0.44 (±0.20) | 0.50 (±0.50) | 0.44 (±0.30) | 0.65 | 0.44 | 0.13 | *F*(2,71) = 0.169; *p* = 0.845 |
| PG | 0.41 (±0.32) | 0.26 (±0.14) | 0.47 (±0.35) | 0.59 | 0.45 | 0.26 | *F*(2,71) = 1.769; *p* = 0.178 |
| PA | 0.40 (±0.22) | 0.49 (±0.29) | 0.36 (±0.28) | 0.27 | 0.67 | 0.86 | *F*(2,71) = 0.919; *p* = 0.403 |
| LPC | 0.22 (±0.08) | 0.21 (±0.09) | 0.19 (±0.07) | 0.24 | 0.24 | 0.25 | *F*(2,71) = 0.619; *p* = 0.541 |
| MhCer | 0.10 (±0.05) | 0.09 (±0.03) | 0.08 (±0.04) | 0.09 | 0.13 | 0.10 | *F*(2,71) = 0.505; *p* = 0.605 |
| LPI | 0.08 (±0.05) | 0.05 (±0.04) | 0.08 (±0.09) | 0.06 | 0.04 | 0.01 | *F*(2,71) = 0.807; *p* = 0.450 |
| LPS | 0.05 (±0.03) | 0.04 (±0.02) | 0.05 (±0.03) | 0.07 | 0.03 | 0.02 | *F*(2,71) = 0.816; *p* = 0.446 |
| dhCer | 0.04 (±0.02) | 0.03 (±0.02) | 0.04 (±0.02) | 0.04 | 0.04 | 0.02 | *F*(2,71) = 0.255; *p* = 0.776 |
| Sulf | 0.04 (±0.03) | 0.05 (±0.05) | 0.03 (±0.02) | 0.04 | 0.05 | 0.03 | *F*(2,71) = 0.728; *p* = 0.487 |
| LPEp | 0.04 (±0.04) | 0.03 (±0.01) | 0.04 (±0.03) | 0.06 | 0.03 | 0.01 | *F*(2,71) = 0.335; *p* = 0.716 |
| GM3 | 0.02 (±0.01) | 0.02 (±0.01) | 0.02 (±0.01) | 0.01 | 0.07 | 0.02 | *F*(2,71) = 0.676; *p* = 0.512 |
| GB3 | 0.02 (±0.01) | 0.02 (±0.007) | 0.02 (±0.02) | 0.01 | 0.03 | 0.02 | *F*(2,71) = 1.010; *p* = 0.369 |
| AcylPG | 0.02 (±0.01) | 0.01 (±0.005) | 0.02 (±0.01) | 0.04 | 0.02 | 0.01 | *F*(2,71) = 2.909; *p* = 0.061 |
| LPCe | 0.009 (±0.004) | 0.009 (±0.004) | 0.01 (±0.005) | 0.01 | 0.01 | 0.006 | *F*(2,71) = 0.048; *p* = 0.953 |
| NSer | 0.003 (±0.002) | 0.002 (±0.001) | 0.004 (±0.004) | 0.01 | 0.003 | 0.001 | *F*(2,71) = 1.295; *p* = 0.280 |

**Supplementary Table 3 - Number of donors per diagnosis**

| Diagnosis | Number of Donors (n = 74) |
| --- | --- |
| Colorectal Cancer Metastasis | 24 |
| Non-digestive system related | 13 |
| Colorectal Cancer | 8 |
| Cholangiocarcinoma | 5 |
| Cyst | 3 |
| Familial Adenomatous Polyposis | 2 |
| Familial Amyloid Polyneuropathy | 2 |
| Primary sclerosing cholangitis | 2 |
| Alcoholic Cirrhosis (resection) | 1 |
| Succinate dehydrogenase deficient (SDH-deficient) gastrointestinal stromal tumor | 1 |
| Adenoma | 1 |
| Alagille Syndrome | 1 |
| Breast Cancer Metastasis | 1 |
| Cryptogenic Cirrhosis | 1 |
| Methylmalonic Acidemia | 1 |
| Focal Nodular Hyperplasia | 1 |
| Gastrointestinal stromal tumor | 1 |
| Hyperoxaluria | 1 |
| Maple syrup urine disease | 1 |
| Mucinous cystic neoplasm | 1 |
| Steatosis | 1 |
| Suspected cholangiocarcinoma | 1 |
| Suspected hepatocellular carcinoma | 1 |

**Supplementary Table 4 – P-values for sex comparisons across the whole sample**

| Lipids | p-value |
| --- | --- |
| FC | 0.680 |
| TG | 0.257 |
| PE | 0.503 |
| CE | 0.881 |
| PS | 0.765 |
| PC | 0.244 |
| PI | 0.385 |
| MG | 0.192 |
| SM | 0.171 |
| DG | 0.853 |
| BMP | 0.527 |
| PEp | 0.596 |
| AC | 0.165 |
| Cer | 0.907 |
| LacCer | 0.150 |
| dhSM | 0.681 |
| PCe | 0.083 |
| LPE | 0.671 |
| PG | 0.641 |
| PA | 0.267 |
| LPC | 0.309 |
| MhCer | 0.687 |
| LPI | 0.722 |
| LPS | 0.991 |
| dhCer | 0.823 |
| Sulf | 0.163 |
| LPEp | 0.588 |
| GM3 | 0.695 |
| GB3 | 0.766 |
| AcylPG | 0.655 |
| LPCe | 0.096 |
| NSer | 0.089 |

**Supplementary Table 5 – P-values for pairwise comparisons across *APOE* genotype and donor sex**

| Lipids | ε3/ε3F vs ε2/ε3F | ε3/ε4F vs ε2/ε3F | ε2/ε3M vs ε2/ε32F | ε3/ε3M vs ε2/ε3F | ε3/ε4M vs ε2/ε3F | ε3/ε4F vs ε3/ε3F | ε2/ε3M vs ε3/ε3F |
| --- | --- | --- | --- | --- | --- | --- | --- |
| FC | 0.911 | 0.911 | 1.000 | 0.911 | 0.911 | 0.911 | 0.911 |
| TG | 0.116 | 0.929 | 0.929 | 0.586 | 0.116 | 0.368 | 0.417 |
| PE | 0.388 | 0.760 | 0.574 | 0.961 | 1.000 | 0.961 | 0.961 |
| CE | 0.810 | 0.810 | 0.810 | 0.982 | 0.810 | 0.810 | 0.810 |
| PS | 0.846 | 0.846 | 0.871 | 0.868 | 1.000 | 0.816 | 0.816 |
| PC | 0.944 | 1.000 | 0.944 | 0.944 | 0.944 | 0.944 | 0.944 |
| PI | 0.776 | 0.776 | 1.000 | 0.776 | 1.000 | 1.000 | 1.000 |
| MG | 0.696 | 0.761 | 0.696 | 0.696 | 0.696 | 0.696 | 0.696 |
| SM | 0.599 | 1.000 | 0.669 | 0.685 | 0.675 | 0.599 | 0.960 |
| DG | 0.777 | 0.802 | 0.809 | 0.809 | 0.426 | 0.446 | 0.777 |
| BMP | 0.704 | 0.704 | 0.802 | 1.000 | 0.704 | 0.704 | 0.911 |
| PEp | 0.930 | 0.930 | 0.930 | 0.930 | 0.930 | 0.930 | 1,000 |
| AC | 0.349 | 0.447 | 0.663 | 0.447 | 0.414 | 0.717 | 0.414 |
| Cer | 0.337 | 0.704 | 1.000 | 0.704 | 0.513 | 0.277 | 0.500 |
| LacCer | 1.000 | 0.929 | 0.929 | 0.929 | 0.929 | 0.983 | 0.929 |
| dhSM | 1.000 | 1.000 | 1.000 | 1.000 | 1.000 | 1.000 | 1.000 |
| PCe | 0.857 | 0.902 | 0.857 | 0.857 | 0.857 | 0.857 | 0.857 |
| LPE | 0.926 | 0.926 | 0.926 | 0.926 | 0.926 | 0.926 | 0.926 |
| PG | 0.871 | 0.902 | 0.871 | 0.871 | 0.871 | 0.871 | 0.871 |
| PA | 0.955 | 1.000 | 0.955 | 0.955 | 0.955 | 0.955 | 0.955 |
| LPC | 0.962 | 0.962 | 0.962 | 0.962 | 0.962 | 0.962 | 0.962 |
| MhCer | 0.563 | 0.563 | 0.871 | 0.563 | 0.248 | 0.973 | 0.563 |
| LPI | 0.945 | 1.000 | 1.000 | 0.945 | 0.951 | 0.945 | 0.945 |
| LPS | 0.959 | 0.131 | 0.661 | 0.439 | 0.853 | 0.105 | 0.439 |
| dhCer | 0.944 | 0.944 | 0.944 | 0.944 | 0.944 | 0.944 | 1.000 |
| Sulf | 0.897 | 0.897 | 0.897 | 0.897 | 0.897 | 0.897 | 0.897 |
| LPEp | 0.749 | 0.749 | 0.749 | 0.749 | 0.749 | 0.749 | 0.908 |
| GM3 | 0.429 | 0.595 | 0.799 | 0.429 | 0.429 | 0.429 | 0.429 |
| GB3 | 0.898 | 0.898 | 0.816 | 0.816 | 0.894 | 0.894 | 0.816 |
| AcylPG | 0.572 | 1.000 | 0.795 | 0.795 | 0.795 | 0.572 | 0.795 |
| LPCe | 0.981 | 0.981 | 0.981 | 0.981 | 0.981 | 0.981 | 0.981 |
| NSer | 0.978 | 0.978 | 0.978 | 0.978 | 0.978 | 0.978 | 0.978 |

Continued on next page

| Lipids | ε3/ε3M vs ε3/ε3F | ε3/ε4M vs ε3/ε3F | ε2/ε3M vs ε3/ε4F | ε3/ε3M vs ε3/ε4F | ε3/ε4M vs ε3/ε4F | ε3/ε3M vs ε2/ε3M | ε3/ε4M vs ε2/ε3M | ε3/ε4M vs ε3/ε3M |
| --- | --- | --- | --- | --- | --- | --- | --- | --- |
| FC | 0.911 | 0.911 | 0.911 | 0.911 | 0.911 | 0.911 | 0.911 | 0.911 |
| TG | 0.248 | 0.417 | 0.939 | 0.929 | 0.162 | 1.000 | 0.248 | 0.116 |
| PE | 0.246 | 0.261 | 0.961 | 0.540 | 0.590 | 0.388 | 0.388 | 0.961 |
| CE | 0.810 | 0.544 | 0.810 | 0.810 | 0.544 | 0.810 | 0.544 | 0.639 |
| PS | 0.816 | 0.846 | 1.000 | 0.846 | 0.846 | 0.846 | 0.846 | 0.846 |
| PC | 0.944 | 0.944 | 0.944 | 0.944 | 0.944 | 0.975 | 1.000 | 0.944 |
| PI | 1.000 | 1.000 | 1.000 | 1.000 | 1.000 | 1.000 | 1.000 | 1.000 |
| MG | 0.696 | 0.696 | 0.696 | 0.696 | 0.696 | 0.696 | 0.696 | 0.867 |
| SM | 0.612 | 0.960 | 1.000 | 0.669 | 0.612 | 1.000 | 0.960 | 0.960 |
| DG | 0.426 | 0.426 | 1.000 | 0.809 | 0.323 | 0.809 | 0.426 | 0.323 |
| BMP | 0.750 | 0.704 | 0.795 | 0.704 | 0.704 | 0.911 | 0.704 | 0.802 |
| PEp | 0.930 | 0.930 | 0.992 | 0.992 | 0.992 | 0.930 | 0.930 | 0.992 |
| AC | 0.493 | 0.663 | 0.663 | 0.708 | 0.663 | 0.663 | 0.414 | 0.414 |
| Cer | 0.277 | 0.983 | 0.704 | 0.513 | 0.277 | 0.778 | 0.704 | 0.500 |
| LacCer | 0.929 | 0.929 | 0.929 | 0.929 | 0.929 | 0.929 | 0.929 | 0.929 |
| dhSM | 1.000 | 1.000 | 1.000 | 1.000 | 1.000 | 1.000 | 1.000 | 1.000 |
| PCe | 0.857 | 0.857 | 0.857 | 0.857 | 0.857 | 0.857 | 0.857 | 0.857 |
| LPE | 0.926 | 0.926 | 0.926 | 0.926 | 0.926 | 0.926 | 0.926 | 0.926 |
| PG | 0.890 | 0.871 | 0.871 | 0.871 | 0.871 | 0.871 | 0.871 | 0.871 |
| PA | 0.955 | 0.955 | 1.000 | 0.955 | 0.955 | 0.975 | 0.955 | 0.955 |
| LPC | 0.962 | 0.962 | 0.962 | 0.962 | 0.962 | 0.962 | 0.962 | 0.962 |
| MhCer | 0.973 | 0.248 | 0.871 | 0.871 | 0.563 | 0.563 | 0.248 | 0.248 |
| LPI | 0.951 | 0.945 | 0.951 | 0.945 | 1.000 | 0.945 | 0.951 | 0.951 |
| LPS | 0.343 | 0.661 | 0.220 | 0.220 | 0.220 | 0.853 | 0.959 | 0.853 |
| dhCer | 0.944 | 0.944 | 0.944 | 1.000 | 0.944 | 0.944 | 1.000 | 0.944 |
| Sulf | 0.897 | 0.897 | 0.897 | 0.897 | 0.897 | 0.897 | 0.897 | 0.897 |
| LPEp | 0.749 | 0.908 | 1.000 | 0.908 | 0.749 | 0.952 | 0.749 | 0.749 |
| GM3 | 0.429 | 0.945 | 0.945 | 0.945 | 0.595 | 0.799 | 0.602 | 0.602 |
| GB3 | 0.816 | 0.816 | 0.816 | 0.894 | 0.898 | 0.933 | 0.898 | 0.898 |
| AcylPG | 0.572 | 0.795 | 0.795 | 0.811 | 0.795 | 1.000 | 1.000 | 0.905 |
| LPCe | 0.981 | 0.981 | 0.981 | 1.000 | 1.000 | 0.981 | 0.981 | 0.981 |
| NSer | 0.978 | 0.978 | 0.978 | 0.978 | 0.978 | 0.978 | 0.978 | 0.978 |

**Supplementary Table 6 – Correlation between hepatic APOE content and lipids**

| Variable | Variable | Rho |  | Prob(Rho) |
| --- | --- | --- | --- | --- |
| Hep APOE (ng/mg total protein) | FC | 0.045 |  | 0.699 |
|  | CE | - 0.129 |  | 0.267 |
|  | AC | - 0.174 |  | 0.132 |
|  | MG | - 0.046 |  | 0.693 |
|  | DG | - 0.202 |  | 0.081 |
|  | TG | - 0.048 |  | 0.684 |
|  | Cer | - 0.245 |  | **0.033** |
|  | dhCer | - 0.197 |  | 0.089 |
|  | SM | 0.092 |  | 0.432 |
|  | dhSM | - 0.061 |  | 0.599 |
|  | MhCer | - 0.114 |  | 0.325 |
|  | Sulf | - 0.015 |  | 0.900 |
|  | LacCer | - 0.318 |  | **0.005** |
|  | GM3 | 0.108 |  | 0.353 |
|  | CB3 | - 0.047 |  | 0.689 |
|  | PA | 0.023 |  | 0.845 |
|  | PC | 0.118 |  | 0.312 |
|  | PCe | 0.041 |  | 0.725 |
|  | PE | 0.113 |  | 0.330 |
|  | PEp | - 0.040 |  | 0.735 |
|  | PS | - 0.061 |  | 0.603 |
|  | PI | 0.130 |  | 0.264 |
|  | PG | - 0.129 |  | 0.266 |
|  | BMP | - 0.081 |  | 0.485 |
|  | AcylPG | - 0.069 |  | 0.554 |
|  | LPC | 0.052 |  | 0.659 |
|  | LPCe | 0.024 |  | 0.839 |
|  | LPE | - 0.060 |  | 0.606 |
|  | LPEp | - 0.282 |  | **0.014** |
|  | LPI | - 0.091 |  | 0.437 |
|  | LPS | - 0.138 |  | 0.233 |
|  | NSer | - 0.233 |  | **0.043** |
|  |  |  |  |  |

Supplementary figure 1

**
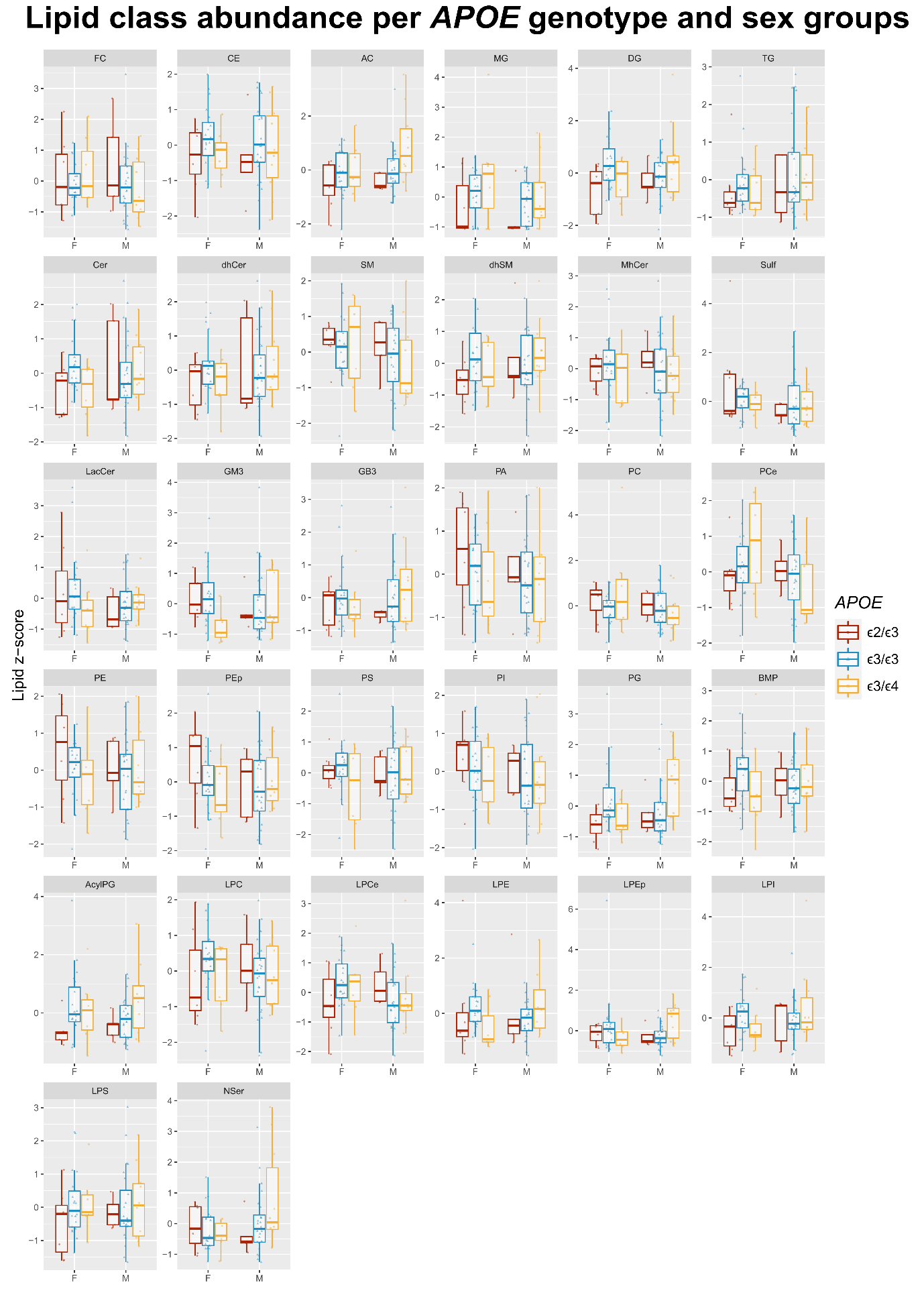
**

Supplementary Figure 2


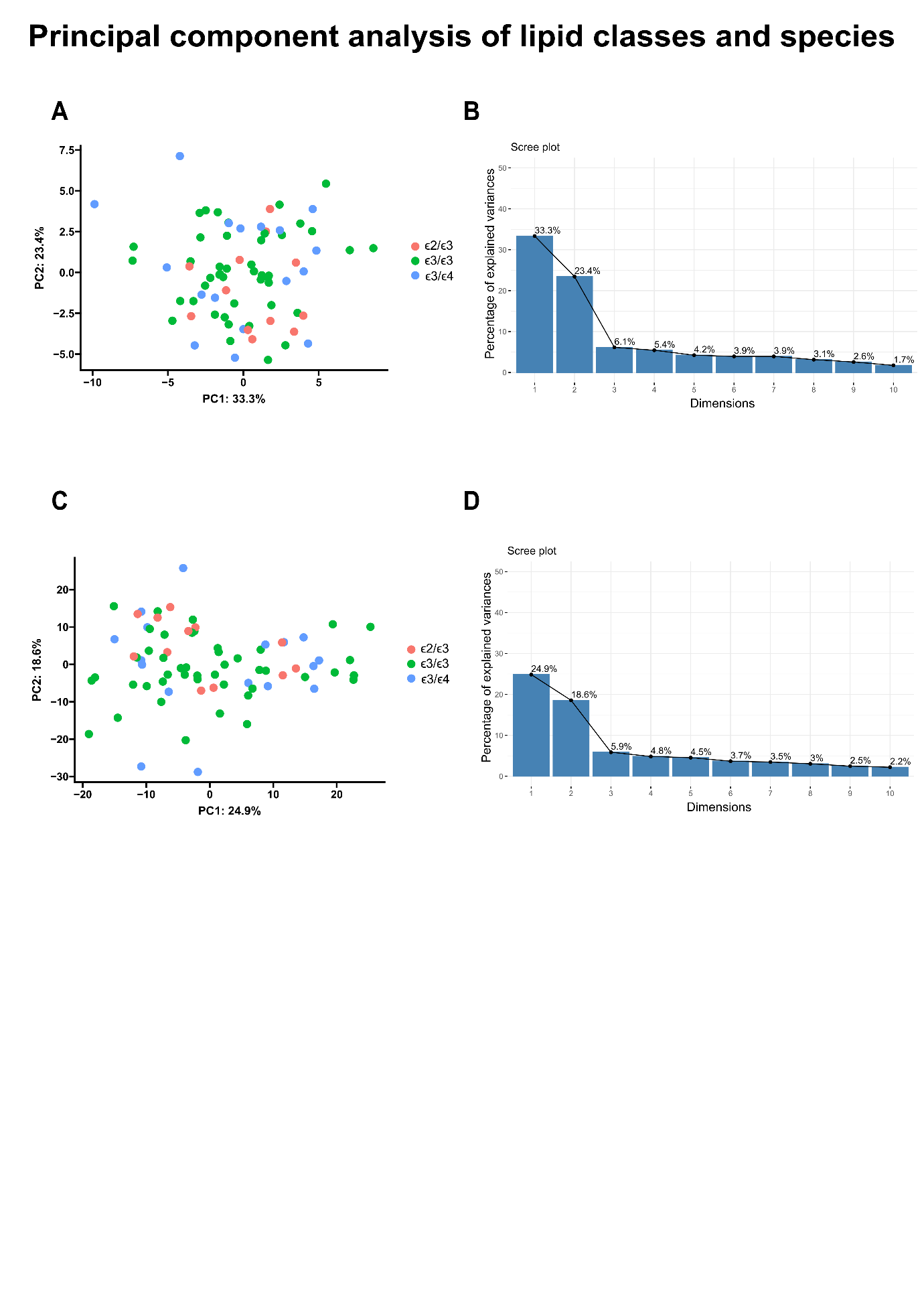

Supplement: Supplementary Information [file mmc1.docx]
